# Supracolloidal Assemblies as Sacrificial Templates for Porous Silk-Based Biomaterials

**DOI:** 10.3390/ijms160920511

**Published:** 2015-08-28

**Authors:** John G. Hardy, Chiara E. Ghezzi, Richard J. Saballos, David L. Kaplan, Christine E. Schmidt

**Affiliations:** 1 J. Crayton Pruitt Family Department of Biomedical Engineering, University of Florida, Biomedical Sciences Building JG-53, P.O. Box 116131, Gainesville, FL 32611-6131, USA; E-Mail: richardsaballos@live.com; 2 Department of Biomedical Engineering, the University of Texas at Austin, Austin, TX 78712, USA; 3 Department of Biomedical Engineering, Tufts University, Medford, MA 02155, USA; E-Mail: Chiara.Ghezzi@tufts.edu

**Keywords:** supramolecular chemistry, supracolloidal chemistry, supramolecular polymers, supramolecular materials, biomaterials, tissue engineering, silk

## Abstract

Tissues in the body are hierarchically structured composite materials with tissue-specific properties. Urea self-assembles via hydrogen bonding interactions into crystalline supracolloidal assemblies that can be used to impart macroscopic pores to polymer-based tissue scaffolds. In this communication, we explain the solvent interactions governing the solubility of urea and thereby the scope of compatible polymers. We also highlight the role of solvent interactions on the morphology of the resulting supracolloidal crystals. We elucidate the role of polymer-urea interactions on the morphology of the pores in the resulting biomaterials. Finally, we demonstrate that it is possible to use our urea templating methodology to prepare *Bombyx mori* silk protein-based biomaterials with pores that human dermal fibroblasts respond to by aligning with the long axis of the pores. This methodology has potential for application in a variety of different tissue engineering niches in which cell alignment is observed, including skin, bone, muscle and nerve.

## 1. Introduction

Bodily tissues are hierarchically structured composite materials with tissue-specific properties that act as cues that dictate the behavior of cells that inhabit them, and such properties can potentially be engineered into instructional tissue scaffolds to achieve similar results [1,2,3,4,5,6]. Topographical control of cell alignment is clearly observable within anisotropically aligned pores that are observed in bone, muscle, nerve and other tissues, which motivates the development of novel methodologies of imparting biomimetic porous structures within biomaterials [1,2,3,4,5,6].

Silk protein-based materials are produced by a number of different species (the most widely studied being that of the domesticated *B. mori* silkworm) [5,7,8,9,10], many of which display interesting mechanical properties and low immunogenicity, and have led to the development of engineered silk-inspired proteins produced recombinantly [11,12,13,14,15], or silk-inspired polymers produced by synthetic chemists [15]. Silk-based biomaterials are popular for a variety of applications because of: (1) their ease of processing in a variety of different solvents (including water, ionic liquids, formic acid, hexafluoroacetone hydrate and hexafluoroisopropanol); (2) the morphologies that can be manufactured (fibers, films, foams, hydrogels); and (3) their ease of chemical modification [7,8,9,10,11,12,13,14,15]. Silk-based biomaterials and their composites are capable of controlled drug delivery, and of acting as cell adhesive tissue scaffolds for a variety of different niches both *in vitro* and *in vivo* [7,8,9,10,11,12,13,14,15].

Although it is possible to employ 3D printing technologies to prepare porous materials, it is challenging to fabricate porous structures accurately mimicking native tissues [1,2,3,4,5,6]. The removal of sacrificial templates (e.g., colloidal crystals, ice crystals, electrospun fibers) from a matrix is an alternative approach that allows the generation of hierarchically organized pores in materials [1,2,3,4,5,6], potentially on the nanoscale [16]. Urea is an inexpensive, non-toxic solid that self-assembles into supracolloidal crystals. The uncontrolled evaporation of water from aqueous solutions of urea yields random networks of dendritic crystals, whereas controlled use of urea seed crystals to initiate crystallization yields relatively well aligned crystals over the length scale of a few hundred micrometers [17]. Zawko and coworkers reported the use of urea to impart pores within photocrosslinkable biopolymer-based hydrogels that typically have mechanical properties similar to soft, and fibroblasts cultured within the gels were observed to align parallel to the fibrillar microstructure of the hydrogels [17]. While entirely aqueous manufacturing processes are appealing, they restrict the selection of materials used to those that are soluble in water (e.g., polysaccharides), and there are many water insoluble polymers used in the clinic. Therefore we used a non-aqueous solvent hexafluoroisopropanol (HFIP) to generate urea-templated polycaprolactone foams [18]. We also reported a simple scalable methodology for aligning the supracolloidal crystals that allows the generation of pores that were aligned over length scales of multiple centimeters within which Schwann cells from the peripheral nervous system aligned [18].

**Figure 1:**
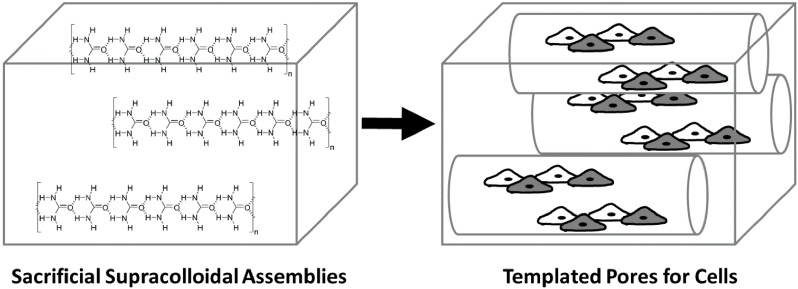
Hydrogen bond mediated self-assembly of supracolloidal assemblies of urea that act as sacrificial templates to impart pores in biomaterials.

In this communication, we explain the solvent interactions governing the solubility of urea which enables us to expand the range of solvents compatible with our urea-based supracolloidal crystal templating methodology, and thereby broadens the scope of polymers it is compatible with. We also highlight the role of solvent interactions on the morphology of the resulting supracolloidal crystals, and moreover, the role of polymer-porogen (silk-urea) interactions on the morphology of the pores in the resulting biomaterials. Finally, we demonstrate that it is possible to use our urea templating methodology to prepare silk protein-based biomaterials with aligned pores that permit cell growth and alignment (Figure 1).

## 2. Results and Discussion

### 2.1. Urea Solubility in Non-Aqueous Solvents

A parameterized approach was used to investigate the ability of non-aqueous solvents to dissolve urea. Solvent–solute interactions play an important role in supramolecular chemistry which has led to quantitative studies of the role of solvents in self-assembly processes [19,20,21,22,23,24]. The bulk properties (e.g., boiling point, density) or molecular level properties (such as specific intermolecular forces) can be quantified and parameterized. Bulk property parameters include the dielectric constant (ε) and Reichardt’s parameter (E_T_, a measure of ionizing power). Molecular level parameters include the Hildebrand solubility parameter, δ (expressed in terms of the total solubility parameter, δ_0_, which is described by the dispersion, polar, and hydrogen bonding parameters, δ_d_, δ_P_, and δ_H_, respectively. The parameters δ_P_ and δ_H_ are described in terms of a combined polar solubility parameter, δ_a_, and the Kamlet-Taft parameters, π* (a generalized polarity parameter), α (the ability to donate hydrogen bonds), and β (the ability to accept hydrogen bonds). The effects of solvents on the hierarchical assembly of supramolecular polymers in non-aqueous solvents have been studied for self-assembling peptides, and while there was a general correlation between the ability of supramolecular polymers to form and the polar solubility parameter, δ_a_ [19,20,21,22,23,24], the precise hydrogen-bonding nature of the solvent in terms of Kamlet-Taft parameters (*i.e.*, deconvolution of the hydrogen bond donors and acceptors) was important to fully understand the solvent effects [19,20,21,22,23,24]. Hydrogen bond donor solvents but not acceptors, played a key role in disrupting amide-mediated self-assembly in non-aqueous solvents.

To generate highly porous biomaterials we require the sacrificial template to be highly soluble in the solvent used during polymer processing. Thus we investigated the ability of a variety of non-aqueous solvents to dissolve urea at a concentration of 100 mg/mL (see Table 1), an arbitrary concentration equivalent to the concentration of polymer, thereby assuring the presence of pores with micrometer scale diameters in the resulting materials.

**Table 1:** The properties of the non-aqueous solvents investigated for the dissolution of urea at a concentration of 100 mg/mL. Solvent parameters: dielectric constant (ε), Reichardt’s parameter (E_T_), and Kamlet-Taft parameters, π*, α, and β. (N.R.) Not reported in the literature; ^a^ Hexafluoroacetone is a gas and it evaporates readily from aqueous solutions; (I.S.) insufficiently soluble; and (S) sufficiently soluble.

Solvent	ε	E_T_	π*	α	Β	Boiling Point (°C)	Surface Tension (mN/m)	Solubility of Urea
Cyclohexane	2.10	0.006	0.00	0.00	0.00	80.7	25.0	I.S.
Toluene	2.38	0.099	0.49	0.00	0.11	110.6	28.5	I.S.
Chloroform	4.80	0.259	0.69	0.44	0.00	61.2	26.7	I.S.
Tetrahydrofuran	7.58	0.207	0.55	0.00	0.55	66.0	26.4	I.S.
Dichloromethane	8.93	0.309	0.73	0.30	0.00	39.6	26.5	I.S.
Ethyl acetate	36.6	0.18	0.55	0.00	0.45	77.1	23.8	I.S.
Acetonitrile	45.60	0.460	0.75	0.19	0.40	82.0	19.1	I.S.
Isopropanol	49.20	0.570	0.48	0.76	0.84	82.6	23.0	I.S.
Butanol	50.20	0.600	0.47	0.84	0.84	117.4	24.2	I.S.
Ethanol	51.90	0.650	0.54	0.86	0.75	78.4	22.3	I.S.
Methanol	55.40	0.760	0.60	0.98	0.66	64.7	22.5	S
Formic acid	57.70	0.830	0.65	1.23	0.38	100.8	37.7	S
Hexafluoroisopropanol	65.30	1.070	0.65	1.96	0.00	58.2	16.1	S
Hexafluoroacetone∙3H_2_O	N.R.	N.R.	N.R.	N.R.	N.R.	−26.0 ^a^	N.R.	S
Water	63.10	1.000	1.09	1.17	0.47	100.0	72.8	S

The solubility of urea in the various solvents was clearly correlated to the parameters describing the bulk properties of the solvent (*i.e.*, the dielectric constant (ε) and Reichardt’s parameter (E_T_)). Indeed, solvents with dielectric constants and Reichardt’s parameters similar to water were the most potent solvents for urea, and there was a threshold of dielectric constants and Reichardt’s parameters below which the urea was insoluble (*ca.* 55 and 0.75, respectively). Interestingly, the Kamlet-Taft polarity parameters provided useful insight into the importance of individual molecular level interactions on the solubility of urea in the respective solvents. There was no clear correlation between the ability of a solvent to dissolve urea and the generalized polarity parameter, π*. While solvents capable of dissolving urea all had π* values of 0.60 or above, this was not a general rule: acetonitrile, chloroform, and dichloromethane (with π* values of 0.75, 0.69 or 0.73 respectively) were poor/non-solvents. There was no correlation between the ability of a solvent to dissolve urea and its ability to accept hydrogen bonds (β). Hexafluoroisopropanol (β = 0) was an excellent solvent for urea whereas cyclohexane (β = 0) was a non-solvent for urea. The Kamlet-Taft parameter that gave the clearest insight into the ability of a solvent to dissolve urea was its ability to donate hydrogen bonds (α), and all of the solvents capable of dissolving urea had α values of ca. 1 or more. Although the solvent parameters for hexafluoroacetone hydrate are not reported in the literature, the acidity of the hydrate (pKa = 6.58) make it a strong hydrogen bond donor capable of dissolving urea [25]. The solvents presented are clearly not an exhaustive list of those capable of dissolving urea at high concentrations, however our parameterized approach to investigating the ability of solvents to dissolve urea should enable others to easily identify other solvents for urea (or analogous sacrificial porogens).

Importantly, the solvents we found to be good solvents for urea (formic acid, hexafluoroisopropanol, hexafluoroacetone hydrate) are suitable for the dissolution of a variety of polymers (including clinically relevant polyesters, peptides, proteins, polyurethanes, *etc.*) which significantly broadens the scope of biomaterials our methodology would be applicable for.

### 2.2. The Role of Solvent Choice on the Morphology of the Sacrificial Supracolloidal Porogens

Of the non-aqueous solvents identified as being capable of dissolving urea (*i.e.*, formic acid, hexafluoroacetone hydrate, hexafluoroisopropanol and methanol) we investigated formic acid, hexafluoroacetone hydrate and hexafluoroisopropanol in more detail (we omitted methanol because it is a non-solvent for silk and known to beta-sheet formation in the silk) [8,9,10,11,12,13,14,15].

Solvent evaporation from aqueous solutions of urea yields dendritic crystals if performed uncontrolled, or less dendritic and relatively well aligned crystals (over the length scale of a few hundred micrometers) if crystallization is initiated in a controlled fashion prior to evaporation using seeds of urea crystals [17]. Thus, we investigated urea crystallization from non-aqueous solvents under both uncontrolled and controlled evaporation conditions. Urea crystallization is controlled by the rate of evaporation of solvent from the solution of urea (100 mg/mL). Uncontrolled urea crystallization experiments were carried out by simply applying urea solutions to the surface of glass microscope slides and allowing the solvent to evaporate. Preliminary experiments attempting to control the directionality of urea crystallization were carried out by sealing the tip of a Pasteur pipette, adding a quantity of urea solution into the Pasteur pipette and allowing the solvent to evaporate slowly from the wide end.

**Figure 2:**
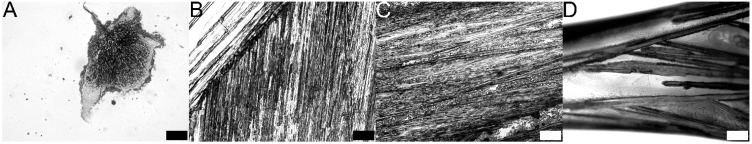
Brightfield microscope image of urea crystals. (**A**) Formed by uncontrolled crystallization from solutions of urea in formic acid (scale bar, 200 μm); (**B**) Formed by uncontrolled crystallization from solutions of urea in hexafluoroacetone hydrate (scale bar, 200 μm); (**C**) Formed by uncontrolled crystallization from solutions of urea in hexafluoroisopropanol (scale bar, 200 μm); and (**D**) Formed by controlled crystallization from solutions of urea in hexafluoroisopropanol in a Pasteur pipette (scale bar, 500 μm).

Of the solvents investigated, formic acid was the solvent with the boiling point and surface tension closest to those of water, and we observed that uncontrolled urea crystallization yielded somewhat dendritic crystals, with sections that were relatively well aligned over the length scale of a few hundred micrometers (Figure 2A). Urea crystals formed from the uncontrolled evaporation of either hexafluoroacetone hydrate (Figure 2B) or hexafluoroisopropanol (Figure 2C) were less dendritic and showed alignment over the length scale of millimeters, although this was not free of defects. Interestingly, controlling the evaporation of hexafluoroisopropanol yielded crystals that were aligned over the length scale of centimeters (Figure 2D). Consequently, we conclude that the solvent-solute interactions govern not only the solubility of the solute, but also the morphology of the supracolloidal assemblies resulting from the evaporation of the solvent.

As the hierarchical supracolloidal assemblies act as sacrificial templates to generate porous biomaterials, we believe that it should be possible to tune the properties of such supracolloidal assemblies in a rational way that will enable the generation of biomaterials instructing cells to assemble in complex biomimetic patterns (e.g., concentric lamellae observed in cortical bone, or helicoidal multi-lamellar alignment of corneal stroma tissue). Recent advances in supramolecular architectonics, particularly DNA-mediated interactions that have programmable structures from the Å to colloidal length scales suggest that we will see the first examples of these in the near future [26,27,28,29,30,31,32]. Moreover, the rational design of the constituent supramolecular building blocks [33,34,35,36,37] offers the prospect of precisely positioning functional species (e.g., nanoparticles) that may deliver therapeutics with precise spatial control, or sense and report changes in the properties of the surrounding tissues (e.g., clusters of nanoparticles whose optical properties change in response to chemical, electrical or mechanical triggers), which may be of use both *in vitro* and perhaps also *in vivo*.

### 2.3. Supracolloidal Templation of Porous Silk Biomaterials

**Figure 3:**
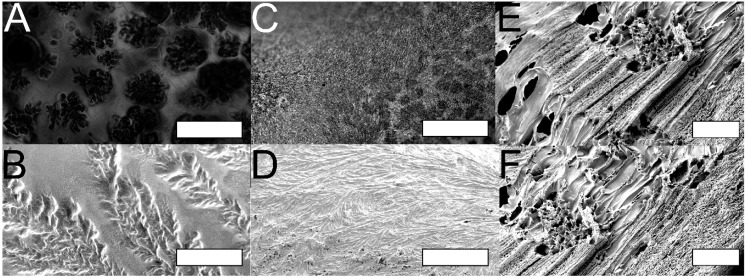
(**A**) Brightfield microscope image of urea crystal-templated silk films from solution in formic acid (scale bar, 600 μm); (**B**) SEM image of urea crystal-templated silk films formed from solution in formic acid (scale bar, 200 μm); (**C**) Brightfield microscope image of urea crystal-templated silk films formed from solution in hexafluoroacetone hydrate (scale bar, 600 μm); (**D**) SEM image of urea crystal-templated silk films formed from solution in hexafluoroacetone hydrate (scale bar, 100 μm); (**E**) SEM image of urea crystal-templated silk films formed from solution in hexafluoroisopropanol (scale bar, 100 μm); and (**F**) SEM image of urea crystal-templated silk films formed from solution in water (scale bar, 100 μm). Images are representative of at least 3 locations on 3 samples.

Urea is a well-known and widely utilized protein denaturant in aqueous solutions, and denatures proteins by disrupting the intermolecular and intramolecular hydrogen bonding interactions that cause proteins to fold or associate into hierarchical assemblies. While the uncontrolled crystallization of urea alone from formic acid yielded somewhat dendritic crystals with sections that were relatively well aligned over the length scale of a few hundred micrometers, the co-crystallization of urea and silk from formic acid, followed by washing to remove the urea, yielded silk-based films with dendritic crystal-templated pores with dimensions of *ca.* 200–400 μm (Figure 3A,B). Likewise, co-crystallization of urea and silk from hexafluoroacetone hydrate followed by washing also yielded silk-based films with very fine dendritic crystal-templated pores with dimensions of approximately 5–10 μm in diameter and up to 100 μm in length (Figure 3C,D). Co-crystallization of urea and silk from either hexafluoroisopropanol (Figure 3E) or water (Figure 3F) followed by washing yielded foams with larger pore diameters (10–40 μm) and lengths extending several hundred micrometers. These results suggest that molecular level interactions between the polymer and sacrificial template (in this case hydrogen bonding interactions between the silk and urea), and bulk solvent parameters (e.g., boiling point) play a role in dictating the morphology of the macroscopic pore structures within biomaterials generated in this fashion. Evidence for which can be observed in the predominantly dendritic pore structures in foams derived from formic acid and hexafluoroacetone hydrate instead of the more linear pore structures in foams derived from hexafluorisopropanol or water. In the future we foresee prospects for tuning the polymer-porogen interactions that will facilitate rational design of pore structure within biomaterials (particularly if DNA-architectonics were employed) [26,27,28,29,30,31,32].

**Figure 4:**
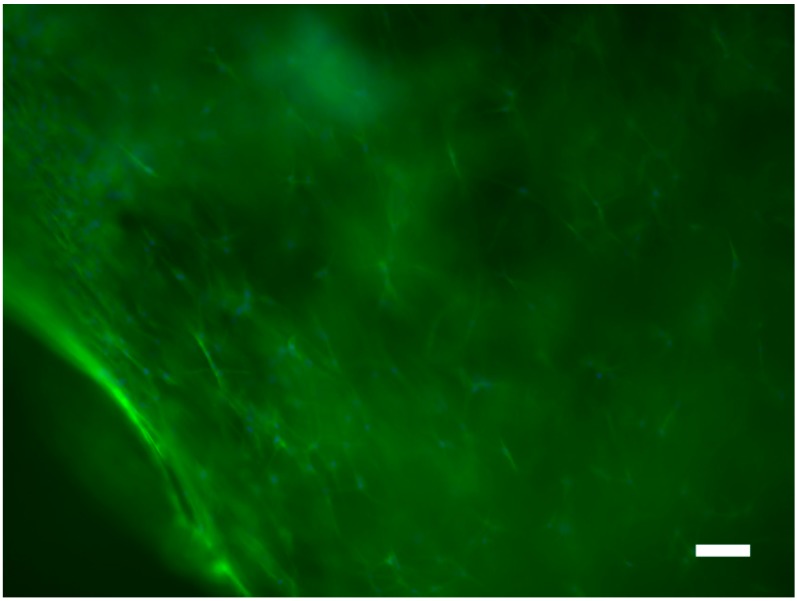
Image of fibroblasts on the surface of the porous silk substrates stained with DAPI (nuclei) and Alexa Fluor 488^®^ Phalloidin (actin filaments); scale bar, 100 μm. Images are representative of at least 3 locations on 3 samples.

Finally, to demonstrate that the pores imparted to silk-based biomaterials by supracolloidal assemblies of urea act as topographical guidance cues for cells cultured inside them, we cultured human dermal fibroblasts in water-derived scaffolds for five days. Obtaining images of the live cells inside the scaffolds using the common cell-permeant dye Calcein AM was challenging because of the strong background fluorescence of the scaffolds, however, it was possible to obtain markedly improved images of the cells inside the scaffolds after fixing them and staining with 4′,6-diamidino-2-phenylindole (DAPI) (nuclei) and Alexa Fluor 488^®^ Phalloidin (actin filaments), and we observed that the fibroblasts preferentially aligned with the pores inside the scaffold (Figure 4).

## 3. Experimental Section

### 3.1. Materials

Unless otherwise stated, all chemicals for synthesis and physicochemical analysis were of American Chemical Society (ACS) grade, purchased from Sigma-Aldrich (St. Louis, MO, USA) and used as received without further purification. *Bombyx mori* silkworm fibroin was purchased from eBay. Reagents for cell culture were purchased from Invitrogen (Carlsbad, CA, USA) unless otherwise noted.

### 3.2. Urea Crystallization from Non-Aqueous Solvents

Urea was added to a non-aqueous solvent at a concentration of 0.1 g/mL. Samples were shaken in airtight containers (typically 15 or 50 mL centrifuge tubes) at 1000 rpm using a Thermomixer C (Eppendorf International, Hauppauge, NY, USA) for 48 h after which they were visually assessed to determine if the urea was soluble. Optically clear solutions were pipetted onto glass microscope slides (width 2.5 cm, length 7.5 cm) using disposable transfer pipettes. The solvent was allowed to evaporate in a fume hood at room temperature for 48 h and then dried under vacuum in a desiccator for 24 to 48 h. Preliminary experiments attempting to control the directionality of urea crystallization were carried out by sealing the tip of a Pasteur pipette, pipetting 200 μL of solution into the vertical Pasteur pipette and allowing the solvent to evaporate slowly from the wide end. Images are representative of at least 3 locations on 3 samples.

### 3.3. Urea Crystal Templating of Porous Silk-Based Films

Silk (2 g) and urea (2 g) were dissolved in either formic acid (20 mL) or hexafluoroacetone trihydrate (20 mL). Samples were shaken in airtight centrifuge tubes (50 mL) at 1000 rpm using a Thermomixer C until the components had fully dissolved (typically 24 to 48 h). Optically clear solutions of urea were pipetted onto glass microscope slides (width 2.5 cm, length 7.5 cm) using disposable transfer pipettes. The solvent was allowed to evaporate in a fume hood at room temperature for 48 h and then dried under vacuum in a desiccator for 24 to 48 h.

Silk-based samples were immersed in aqueous methanol (80% methanol) for 1 h to assure that the silk was rendered water insoluble due to the formation of inter- and intra-molecular β-sheets, and then placed in a container of water to wash out urea and methanol. The samples were washed with water for 3 days to remove any traces of urea, typically exchanging the water every 3 h (*i.e.*, *ca.* 24 times), after which they were dried under high vacuum for 24 to 48 h.

### 3.4. Preparation of Silk-Based Tissue Scaffolds with Aligned Pores

Solutions of silk and urea in hexafluoroisopropanol were prepared as described in Section 2.2. Polydimethylsiloxane (PDMS) templates [18] were placed on flat rigid surfaces, and solutions of silk/urea were pipetted into the grooves using disposable transfer pipettes. Glass microscope slides (width 2.5 cm, length 7.5 cm) were placed on top of the PDMS templates and the solvent was allowed to evaporate (typically 144 h), after which the slides were removed and the silk/urea composites were then dried under vacuum in a desiccator for 24 to 48 h. Samples were immersed in aqueous methanol (80% methanol) for 1 h to assure that the silk was rendered water insoluble due to the formation of inter- and intra-molecular β-sheets, and then placed in a container of water to wash out urea and methanol. The samples were washed with water for 3 days to remove any traces of urea, typically exchanging the water every 3 h (*i.e.*, *ca.* 24 times), after which they were dried under high vacuum for 24 to 48 h. The resulting white silk-based tissue scaffolds had thicknesses of ca. 0.4 mm as determined using high precision digital calipers (ThermoFisher Scientific, Waltham, MA, USA), widths of *ca.* 2 mm, and pores aligned over lengths of up to 0.5 cm. Samples were cut to lengths appropriate for the various subsequent experiments using a razor blade.

### 3.5. Optical Microscopy of Urea and Silk Materials

Brightfield images of crystals of urea cast from formic acid, hexafluoroacetone hydrate and hexafluoroisopropanol, or porous silk-based materials were obtained using an Olympus IX70 inverted microscope (Olympus Corporation of the Americas Inc., Center Valley, PA, USA) equipped with an Olympus DP80 dual color and monochrome digital camera (a 1.4 megapixel Bayer mosaic color CCD camera) that was attached to the microscope with a 0.63 B-mount. Image Analysis was performed using Olympus cellSens^®^ imaging software, Version 1.11 (Olympus Corporation of the Americas Inc.). Images are representative of at least 3 locations on 3 samples.

### 3.6. Scanning Electron Microscopy (SEM)

Images of porous silk-based materials obtained using a scanning electron microscope (SEM). Samples were mounted on a SEM stub and sputter coated with Pt/Pd (15 nm) using a Cressington 208 benchtop sputter coater (Cressington Scientific Instruments, Watford, UK). All samples were imaged using a Zeiss Supra 40 VP field emission scanning electron microscope. Images are representative of at least 3 locations on 3 samples.

### 3.7. In Vitro Cell Culture

Human dermal fibroblasts (HDF, Invitrogen) were cultured in high glucose Dulbecco’s Modified Eagle Medium (DMEM) supplemented with GlutaMAX™ Supplement (Invitrogen), 10% fetal bovine serum (FBS), and 1% penicillin/streptomycin antibiotic (Invitrogen). Cells were maintained at 37 °C in humidified atmosphere of 5% CO_2_. HDF were seeded on the silk scaffold after treatment in ethanol for 1 h. Samples were subsequently rinsed three times in PBS and incubated in complete media before seeding. HDF were seeded at a density of 50,000 cells/cm^2^ and cultured for 1 week. Calcein AM staining of live cells was applied in accordance with the instructions supplied with the kit (Invitrogen).

To stain the actin filaments and nuclei within cells, HDF were fixed in 2.5% paraformaldehyde in PBS at room temperature for 20 min, and then rinsed in PBS three times for 5 min. Cells were permeabilized in ice cold acetone (Sigma) at −20 °C for 5–10 min, and then rinsed in PBS three times. Samples were incubated in 4′,6-diamidino-2-phenylindole (DAPI) solution at 300 nM for 10 min and for 1 h in 1:1000 Alexa Fluor^®^ 488 Phalloidin (Invitrogen), followed by rinsing. Samples were imaged with a Keyence Fluorescence Microscope BZ-X700 (Keyence Corporation of America, Itasca, IL, USA) with excitation and emission filters at 495–518 nm for Alexa Fluor^®^ 488 and 358–461 nm for DAPI. Images are representative of at least 3 locations on 3 samples.

## 4. Conclusions

Porous biomaterials are widely used in drug delivery and tissue engineering. 3D printing technologies are very promising for the preparation of porous biomaterials, however, they tend to be expensive. The removal of sacrificial templates (e.g., colloidal crystals, ice crystals, fibers) from a matrix is a comparatively low cost alternative that allows the generation of hierarchically organized pores in biomaterials. Herein, we expand our studies on the use of sacrificial supracolloidal templates for the generation of porous biomaterials. We expand the scope of polymers that the methodology is compatible with by elucidating the solvent interactions governing the solubility of urea. We highlight the role of solvent interactions on the morphology of the resulting supracolloidal crystals. We also highlight the role of polymer-urea interactions on the morpohology of the pores in the resulting biomaterials. Finally, we demonstrate that it is possible to use our urea templating methodology to prepare *B. mori* silk protein-based biomaterials with pores that human dermal fibroblasts respond to by aligning with the long axis of the pores. We believe that our methodology has potential for application in a variety of different tissue engineering niches in which cell alignment is observed, including skin, bone, muscle and nerve; particularly when combined with the potential to guide the directionality of the urea crystals over multiple centimeters [18].
